# Editorial for the Special Issue on ‘Application and Behavior of Nanomaterials in Water Treatment’

**DOI:** 10.3390/nano9060880

**Published:** 2019-06-14

**Authors:** Protima Rauwel, Wolfgang Uhl, Erwan Rauwel

**Affiliations:** 1 Institute of Technology, Estonian University of Life Sciences, Kreutzwaldi 56/1, 51014 Tartu, Estonia; erwan.rauwel@emu.ee; 2 Norwegian Institute for Water Research (NIVA), Gaustadalléen 21, N-0349 Oslo, Norway; wolfgang.uhl@niva.no; 3 Department of Civil and Environmental Engineering, Norwegian University of Science and Technology (NTNU), S. P Andersens Vei 5, 7491 Trondheim, Norway

The simultaneous population explosion and the growing lack of clean water today requires disruptively innovative solutions in water remediation. The last decade has witnessed the emergence of various nanomaterials capable of bridging the gap between the demand for and supply of clean water. Accelerated research on finding suitable nanomaterials in water treatment is therefore fueled by the need of the hour. The main asset of nanomaterials is their highly specific surfaces due to their size reduction, which in turn promotes enhanced catalytic activity, subsequently bringing about a more efficient degradation of dyes and organic pollutants. Nanomaterials such as oxide nanoparticles, nanocarbons, doubled layered hydroxides, and other nanosorbents offer enormous advantages in heavy metal capture and extraction from aqueous media.

This Special Issue compiles eleven articles dedicated to nanomaterials for water treatment: ten research articles and one review article. Together they constitute an interesting and a multi-disciplinary approach to pollution elimination in aqueous media. The papers present different nanomaterials such as layered double hydroxides [1]; nanoporous carbon [2]; oxide nanoparticles, i.e., ZnO [3] and MnO [4]; Ag metal nanoparticles [5]; polymer fibers [6,7]; and inorganic BiOCl doped Dy^+3^ powders [8]. Hybrid materials combining metal organic frameworks (MOF) such as MIL-88A [9] and Prussian blue combined with graphene and carbon nanotubes (CNT) [10], along with magnetic nanoparticles, i.e., magnetite (Fe_3_O_4_) and ferrite (Mn-Zn) [11], are also featured. These nanomaterials have been applied to the degradation of dyes and pharmaceuticals, along with heavy metal ion and radioactive ion extraction. The purpose of this Special Issue is to communicate the most recent advances in the application and behavior of nanomaterials in water treatment. It targets a broad readership of physicists, chemists, materials scientists, catalysis researchers, water researchers, environmentalists, and nanotechnologists. In the paragraphs that follow, we, the guest editors of this Special Issue, provide a brief overview of the individual articles published and hope to incite the interest of potential readers. 

We open the discussion on the published articles with the paper on silver metal nanoparticles by Shim et al. [5]. Their work focuses on desalination via the extraction of radioactive iodine from water. Their methodology combines silver nanoparticles immobilized on a cellulose-based membrane reinforced with *Deinococcus radiodurans*, which is a radiation-resistant bacterium. Ag nanoparticles capture iodine complexes, whereas the bacteria serve to bio-remediate the produced slurry. Metal oxide nanoparticles have also been presented in this compilation for the degradation of organic species, dyes, and pharmaceuticals. The study by Khan et al. assesses the effects of ZnO nanoparticles in various contaminated aqueous media [3]. Their study is of importance, as ZnO nanoparticles are employed in various applications, and therefore their concentrations in wastewaters are increasing. They more specifically study the stability of ZnO in the presence of persistent organic pollutants, i.e., polybrominated diphenyl ethers. The latter behaves as a surfactant tending to increase the colloidal stability of ZnO nanoparticles, which could prove detrimental if consumed. Other inorganic nanomaterials for the degradation of methylene blue were studied by Xu et al. [4]. They synthesized MnO nanomaterials with rod-like morphologies, which have the potential to be reused in successive cycles against methylene blue degradation with a high efficiency of 99.8%. Inorganic powders of BiOCl were doped with Dy^+3^ by Yang et al. [8]. Their paper describes the synthesis of the rare-earth doped inorganic nanopowders and their photocatalytic activity towards Rhodamine B degradation. The authors have worked on the structural, optical, and adsorption properties of the nanopowder. They have also provided a schematic of the energy band diagram and electron transfer mechanism in the Dy^+3^ doped and undoped BiOCl powders.

Organic materials such as polymer fibers also demonstrate efficacy in degradation of dyes and pharmaceuticals. The report by Guo et al. describes a cost-effective and facile fabrication of electrospun ε-polycaprolactone- and β-cyclodextrin-based composite polymer fiber [7]. These fibers exhibit high surface areas, improved mechanical strength, and excellent uptake of methylene blue azo dye. Wang et al. have also used polycaprolactone fibers modified by polydopamine [6]. The nanocomposite had a roughened microstructure, implying a higher specific surface with more active sites for the extraction of dyes. They exhibited efficiency against both methylene blue and methylene orange. Other organic materials include activated carbons, which have also presented efficiency against dyes such as methylene blue. Shi et al. have synthesized nanoporous carbon from metal organic complexes [2]. Their tunable pore sizes and uniform pore distribution allow diffusion of the methylene blue molecules through them. Nanoporous carbons are excellent electrode materials and exhibit supercapacitance properties in aqueous electrolytes. Due to their higher anion exchange capacity, layered double hydroxides (LDH) are considered to be promising nanomaterials for the extraction of organic and inorganic anions [1]. Wang et al. have synthesized Ni-Al-Fe LDH, which exhibited a higher photocatalytic activity than pure LDH. Their study demonstrates a catalytic effect of the captured heavy metals on the surface of LDH towards the degradation of organic contaminants in wastewater.

Magnetic extraction has the advantage of reclaiming the spent sorbent. Fe_3_O_4_-MIL-88A is one such magnetic MOF presented by Liu et al., capable of degrading phenolic dyes, i.e., bromophenol blue [9]. They tested their material’s efficiency on nine dyes in all, out of which eight contained sulphonyl groups. Their study therefore brings insights into magnetic extraction of dyes. Magnetic photocatalysts, i.e., BiVO_4_/Mn_1-x_Zn_x_Fe_2_O_4_/RGO, were studied by Xie et al. in their work [11]. They thoroughly investigated the photocatalytic activity of the composite. In addition, graphene played a very important role in enhancing the photocatalytic activity of the material towards the degradation of Rhodamine B dye. The last paper by Rauwel et al. is a contribution from the guest editors, and reviews the various hybrid nanomaterials studied by various groups [10]. They focus on the extraction of ^137^Cs^+^ from aqueous media in the light of the recent Fukushima Daiichi catastrophe. The paper mainly surveys the extraction of ^137^Cs^+^ with nanocomposites of Prussian blue/graphene/CNT. The possibility of magnetic extraction when combining the hybrid material with Fe_3_O_4_ nanoparticles is also discussed. 
